# Optimal timing of cranioplasty after post-injury decompressive craniectomy: a case control study

**Published:** 2012-11

**Authors:** Hosseinali Khalili, Ali Razmkon

**Affiliations:** ^*a*^Department of Neurosurgery, Shiraz University of Medical Sciences, Shiraz, Iran.

## Abstract

**Background:**

Cranioplasty after postinjury decompressive craniectomy (DC) is routinely performed with a three-month delay to avoid the risk of infection and other complications. Recent experience suggests that performing Cranioplasty surgery at shorter period than three months following DC not only may not cause more infections, but also has the privilege of easier dissection, less bleeding, and reduced costs. The present study was aimed at evaluating the optimal timing of cranioplasty by comparing different parameters using two different time frames.

**Methods:**

A total of ninety-five patients underwent cranioplasty surgery during March 2010 to March 2011 in Rajaee Hospital affiliated to Shiraz University of Medical Sciences (Shiraz, Iran). All of them underwent DC surgery because of post traumatic intracranial hypertension. All of patients were divided into two groups with respect o the time period between cranioplasty and DC. For one group this period was 2 months and the other was higher. All relevant demographic and clinical data as well as operative variables such as length of operation, amount of bleeding (post-op Hb drop) and late prognosis were compared between these two groups.

**Results:**

Mean age was 32.2 ± 13.3(SD) years, and 92.6% of patients were male. No significant difference was observed in independent parameters between the two groups with respect to the length of operation (p=0.004) and amount of bleeding (p=0.013) decreased significantly in patients operated earlier than two months from their DC. No significant difference was observed in postoperative complications and final 6 months prognosis.

**Conclusions:**

Findings of the present study showed that performing cranioplasty earlier than two months following craniectomy was associated with shorter surgical duration and lower amounts of bleeding. Performing of cranioplasty in shorter time is accompanied by an easier dissection with no more complications.

**Keywords:**

Cranioplasty, Decompressive, Craniectomy, TBI


					Volume 4, Suppl. 1 Nov 2012
Publisher: Kermanshah University of Medical Sciences
URL: http://www.jivresearch.org
Copyright:
Congress of Iranian Neurosurgeons, 14-16 November 2012, Kermanshah, Iran
Paper No. 13
Optimal timing of cranioplasty after post-injury decompressive
craniectomy: a case control study
Hosseinali Khalili a,*
, Ali Razmkon a
a
Department of Neurosurgery, Shiraz University of Medical Sciences, Shiraz, Iran.
Abstract:
Background: Cranioplasty after postinjury decompressive craniectomy (DC) is routinely performed with a three-
month delay to avoid the risk of infection and other complications. Recent experience suggests that performing
Cranioplasty surgery at shorter period than three months following DC not only may not cause more infections, but
also has the privilege of easier dissection, less bleeding, and reduced costs. The present study was aimed at
evaluating the optimal timing of cranioplasty by comparing different parameters using two different time frames.
Methods: A total of ninety-five patients underwent cranioplasty surgery during March 2010 to March 2011 in
Rajaee Hospital affiliated to Shiraz University of Medical Sciences (Shiraz, Iran). All of them underwent DC surgery
because of post traumatic intracranial hypertension. All of patients were divided into two groups with respect o the
time period between cranioplasty and DC. For one group this period was 2 months and the other was higher. All
relevant demographic and clinical data as well as operative variables such as length of operation, amount of
bleeding (post-op Hb drop) and late prognosis were compared between these two groups.
Results: Mean age was 32.2  13.3(SD) years, and 92.6% of patients were male. No significant difference was
observed in independent parameters between the two groups with respect to the length of operation (p=0.004) and
amount of bleeding (p=0.013) decreased significantly in patients operated earlier than two months from their DC.
No significant difference was observed in postoperative complications and final 6 months prognosis.
Conclusions: Findings of the present study showed that performing cranioplasty earlier than two months following
craniectomy was associated with shorter surgical duration and lower amounts of bleeding. Performing of cranioplasty
in shorter time is accompanied by an easier dissection with no more complications.
Keywords:
Cranioplasty, Decompressive, Craniectomy, TBI
* Corresponding Author at:
Hosseinali Khalili: Shiraz Neuroscience Center, Department of Neurosurgery, SUMS, Shiraz, Iran, Tel: 09173174156,
Email: khalili_h@sums.ac.ir, (Khalili H.).

				

